# P086: Prospective surveillance from the laboratory of multidrug-resistant bacteria (MDRB) bacteremia

**DOI:** 10.1186/2047-2994-2-S1-P86

**Published:** 2013-06-20

**Authors:** NM Dia, A Ndir, R Ka, KL Onanga, ML Dia, B Ndoye, AI Sow, M Seydi

**Affiliations:** 1 Infectious Diseases Department, Fann Teaching Hospital, Dakar, Senegal; 2 Pronalin, Ministry of Health, Dakar, Senegal; 3 Laboratory of Bacteriology, Fann Teaching Hospital, Dakar, Senegal

## Introduction

The control of the diffusion of the MDRB in health establishments is a priority.

## Objectives

Our work studied the incidence of bacteremia due to MDRB in a hospital environment.

## Methods

Microbiological surveillance was led in three departments of a teaching hospital during a period of six months going from April till October 2012 and concerning only blood cultures with diagnostic aim.

## Results

During the study period, 123 patients were followed and 30 episodes of bacteremia were described that is 21 % of all the blood cultures taken. The average age of the patients was 49 years±18.31 and the sex-ratio 0.66. The majority of the patients (78.6 %) were sent by a health care structure, 14.3 % came from the place of residence and 7.1 % were the object of internal transfer. Thirty six percent of the patients were admitted for a neurological disorder with an average duration of stay of 19.66 days ±14.62. Positive blood cultures were attributed to nosocomial infections in 22 cases (75.9 %) with an average delay of acquisition of 14.55 days. On the bacteriological plan, the responsible microorganisms were established: Enterobacteriaceae17 (56.7 %) among which 11 (64.7 %) producing extended-spectrum beta-lactamases (ESBL), non fermenting Gram- negative bacteria 4 (13.3 %), Gram-positive bacteria 9 (30 %) among which 4 methicillin-resistant *Staphylococcus aureus* (MRSA). Klebsiella pneumoniae was the dominant microorganism 8 (26.7 %). The presence of a catheter was identified in 7 cases of bacteremia associated with an Enterobacteriaceaeproducing ESBL. The rate of attack MDRB was 1.54 for 100 admissions and the incidence rate was 1.66 for 1000 patient-days. After bacteremia, the death rate was 61.5 %.

## Conclusion

The incidence of the MDRB during bacteremia is high in our structure. A program of prevention of the diffusion of the MDRB should be set up, accompanied with a training of nursing staff.

## Disclosure of interest

None declared.

